# Comparative Transcriptome Analysis of *Pueraria lobata* Provides Candidate Genes Involved in Puerarin Biosynthesis and Its Regulation

**DOI:** 10.3390/biom13010170

**Published:** 2023-01-13

**Authors:** Huiting Xi, Yaru Zhu, Wenwen Sun, Nan Tang, Zhengqin Xu, Xiaohong Shang, Yansheng Zhang, Huabing Yan, Changfu Li

**Affiliations:** 1 Shanghai Key Laboratory of Bio-Energy Crops, Research Center for Natural Products, Plant Science Center, School of Life Sciences, Shanghai University, Shanghai 200444, China; 2 Key Laboratory of Plant Germplasm Enhancement and Specialty Agriculture, Wuhan Botanical Garden, Chinese Academy of Sciences, Wuhan 430074, China; 3 Cash Crops Research Institute, Guangxi Academy of Agricultural Sciences, Nanning 530007, China

**Keywords:** *Pueraria lobata* (wild), *Pueraria lobata* var. thomsonii, puerarin, *C*-glucosyltransferase, MYB transcription factor

## Abstract

*Pueraria lobata* is a traditional Chinese herb in which an isoflavone *C*-glucoside, namely puerarin, has received the utmost interest due to its medicinal properties. To date, the biogenesis of puerarin, especially its *C*-glucosyl reaction in the pathway, remains poorly understood. Moreover, the transcription factors (TFs) that regulate puerarin biosynthesis in *P. lobata* have not been reported. Here, we performed phytochemical studies on the different developmental stages of the root, stem, and leaf tissues of two *P. lobata* cultivars, which suggested that both the roots and stems of *P. lobata* were the sites of puerarin biosynthesis. RNA-sequencing was conducted with the root and stem tissues of the two cultivars under different stages, and the clean reads were mapped to the recently published genome of *P. lobata* var. thomsonii, yielding the transcriptome dataset. A detailed analysis of the gene expression data, gene coexpression network, and phylogeny proposed several *C*-GTs that likely participate in puerarin biosynthesis. The first genome-wide analysis of the whole MYB superfamily in *P. lobata* presented here identified a total of 123 nonredundant *PlMYB* genes that were significantly expressed in the analyzed tissues. The phylogenetic analysis of PlMYBs with other plant MYB proteins revealed strong PlMYB candidates that may regulate the biosynthesis of isoflavones, such as puerarin.

## 1. Introduction

*Pueraria lobata*, also named kudzu, is a perennial climbing vine of the *Leguminosae* family. This species has long been used as a Chinese traditional medicine for the treatment of cardiovascular problems, alcohol abuse, diabetes, and many other diseases [1]. Extensive phytochemical studies have been conducted with the *Pueraria* genus [2] following the discovery of an important isoflavone, 8-*C*-glucoside, called puerarin in the late 1950s [3]. A variety of bioactivities, such as the prevention of cardiovascular diseases [4], the improvement of bone health [5], antitumor activities [6], and antiviral activities [7], have been displayed by puerarin.

**Figure 1:**
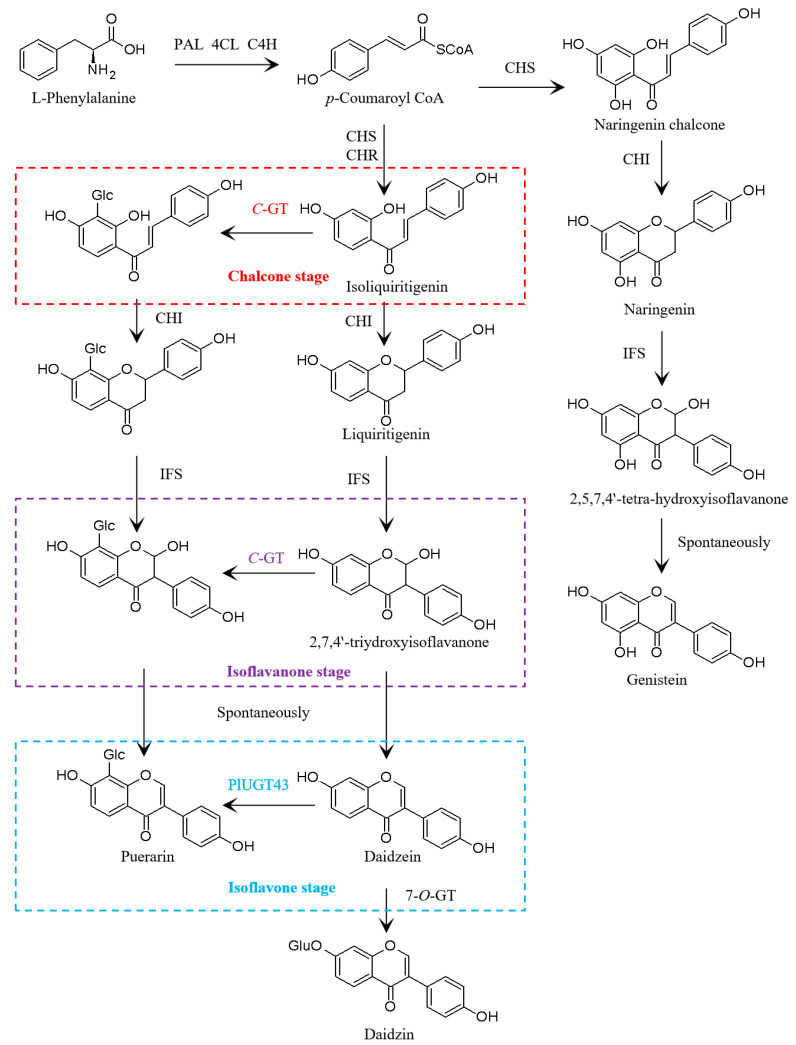
The proposed pathway for the biosynthesis of puerarin. PAL represents phenylalanine ammonia-lyase, C4H represents cinnamate 4-hydroxylase, 4CL represents 4-coumarate:CoA ligase, CHS represents chalcone synthase, CHI represents chalcone isomerase, CHR represents chalcone reductase, IFS represents isoflavone synthase, and *C*-GT represents *C*-glucosyltransferase. *C*-glucosylation during puerarin biosynthesis may take place at the chalcone, isoflavanone, or isoflavone stage. At the isoflavone stage, PlUGT43 catalyzes 8-*C*-glucosylation, converting daidzein to puerarin [10].

Despite the medicinal importance of puerarin, the pathway leading to puerarin, especially the enzymes that catalyze *C*-glucosyl bond formation, is still partially understood. As it is an isoflavonoid, the skeletal formation of puerarin shares a common pathway with other isoflavones. Starting from the aromatic amino acid l-phenylalanine, it is converted to daidzein and/or genistein under the activities of phenylalanine ammonia-lyase (PAL), cinnamate 4-hydroxylase (C4H), 4-coumarate:CoA ligase (4CL), chalcone synthase (CHS), chalcone reductase (CHR), chalcone isomerase (CHI), and isoflavone synthase (IFS) [8,9] (Figure 1).

For the *C*-glucosylating reaction, the previously published data regarding the stage at which the *C*-glucosyl moiety is introduced remain contradictory [10,11,12]. An early labeling study has suggested isoliquiritigenin at the chalcone stage to be the *C*-glucosyl acceptor (Figure 1) [11]. However, enzyme assays using the *Pueraria* roots implied that the *C*-glucosyl bond is formed at the isoflavanone stage using 2-hydroxyisoflavanone as an acceptor (Figure 1) [12]. On the contrary, the recent molecular cloning and characterization of the isoflavone *C*-GT (namely PlUGT43) seemed to support that the *C*-glucosylation reaction in puerarin biosynthesis could take place at the isoflavone stage using daidzein as a substrate (Figure 1) [10,13]. However, this latter conclusion is still challenged by the fact that the catalytic efficiency of the 8-*C*-glucosylation activity (K_at_/K_m_ = 0.01 × 10^4^ × S^−1^M^−1^) [10] of PlUGT43 toward daidzein in puerarin biosynthesis is 370 times lower than the parallel 7-*O*-glucosylation activity (K_at_/K_m_ = 3.7 × 10^4^ × S^−1^M^−1^) [14] of PlUGT1 toward daidzein in daidzin biosynthesis, contrasting with the metabolite profile of more puerarin than daidzin accumulating in the *P. lobata* roots [15]. Thus, further experiments are needed to clarify this inconsistency, and this would largely depend on the systematic identification and characterization of more UGTs in *P. lobata*. On the other hand, understanding the regulation of puerarin biosynthesis may help to increase its yield in *P. lobata* plants, yet none of the transcription factors (TFs) that regulate puerarin biosynthesis were previously identified in *P. lobata*. Considering that several MYB TFs were previously confirmed to play a regulatory role in the isoflavonoid metabolism in *Glycine max* [16,17], which has the closest relationship to *P. lobata* [18], this study focused on the analysis of *P. lobata* MYB TFs.

A transcriptome analysis is an efficient methodology to identify the genes involved in the biosynthesis of plant secondary metabolites. With the aim to identify the *C*-GT in puerarin biosynthesis, this strategy was previously applied to the different tissues of *P. lobata* [19] and to different *Pueraria* species [12,13]. However, all these previous transcriptome analyses depended on the de novo assembly tools, which, to some extent, make getting accurate transcripts challenging [20]. In this study, benefitting from the recently published 1.37 Gb genome assembly of the *P. lobata* var. thomsonii [18], we performed a reference-genome-based assembly of the RNA sequencing-generated reads derived from the different developmental stages of the root and stem tissues of two *P. lobata* varieties. A detailed analysis of the gene expression data, metabolite profile, and phylogenetic relationship allowed us to propose puerarin-related genes, including several *C*-UGT candidates and MYB TFs, that are likely to be involved in the biosynthesis and regulation of puerarin production in *P. lobata*.

## 2. Materials and Methods

### 2.1. Plant Materials and Chemicals

Seeds of *P. lobata* (wild) and *P. lobata* var. thomsonii were obtained from the Guangxi Academy of Agricultural Sciences, China. The two *P. lobata* cultivars were phenotypically indistinguishable in their above ground parts, but the roots of the thomsonii cultivar swelled faster relative to the wild cultivar during the developmental process [18]. The *P. lobata* seeds were sterilized and germinated as previously described [14]. The germinated seedlings with a similar growth size were planted in a soil pot in a growth chamber under a 16/8 h photoperiod at 25 °C (24 h). Roots, stems, and leaves were harvested at different growth stages and stored in liquid nitrogen until use. The chemical standards of puerarin (CAS no. 3681-99-0), daidzein (CAS no. 486-66-8), and genistein (CAS no. 446-72-0) were purchased from Yuanye Co., Ltd. (Shanghai, China).

### 2.2. Phytochemical Analysis

Plant materials were ground in liquid nitrogen with a mortar and pestle and were dried to a constant weight at 37 °C. Twenty milligrams of dried powder was exactly weighed and extracted with 1 mL of methanol under sonication (180 W, 40 kHz, 30 °C, 20 min). The clear methanol extracts were obtained through centrifugation and were directly used for HPLC analysis. For each developmental stage at which the plant materials were sampled, three biological replicates with three technical repeats were performed.

HPLC analysis was performed on an LC-20AT instrument (Shimadzu, Kyoto, Japan). An inertsil ODS-SP reverse phase column (250 mm × 4.6 mm, 5 µm) was used as the chromatographic column with its temperature set at 25 °C. Mobile phases consisted of 0.1% formic acid in water (A) and HPLC-grade acetonitrile (B), and the samples were separated under the following program: 0–5 min at 15–50% of B, 5–35 min at 50–85% of B, 35–40 min at 85% of B, 40–45 min at 85–15% of B, and 45–50 min at 15% of B. The flow rate was 0.8 mL/min, and the monitoring wavelength was set to 250 nm.

### 2.3. RNA Extraction and Transcriptome Sequencing

Total RNA was isolated from the plant materials using a plant RNA Prep Kit (Tiangen Biotech, Beijing, China) following the product manual. The integrity and quantity were determined using an Agilent 2100 Bioanalyzer (Agilent Technologies, Palo Alto, CA, USA). The well-qualified total RNA was sent to the Shanghai Majorbio Bio-pharm Technology Co., Ltd. (Shanghai, China), where the cDNA libraries were constructed, and RNA-sequencing was performed on an Illumina NovaSeq6000 platform. Raw reads were trimmed and quality controlled using fastp (https://githum.com/OpenGene/fastp) [21] using default parameters. To monitor sequencing quality, the values of Q20 and Q30 of the clean data were determined.

### 2.4. Mapping to the P. lobata Genome and Data Analysis

The clean reads were aligned to the reference genome [18] with an orientation mode using HISAT2 software (http://ccb.jhu.edu/software/hisat2/index.shtml) [22]. The mapped reads were then used to reconstruct the transcript in a reference-based approach using StringTie software (https://ccb.jhu.edu/software/stringtie) [23]. Gene function was annotated through BLASTx (E-value < 1 × 10^−5^) search against NR, KOG/COG, Swiss-Prot, KO (KEGG Ortholog), Pfam, and GO databases. The expression of all genes was normalized to fragments per kb per million fragments (FPKM) [24]. The gene coexpression network was established using the WGCNA package [25] installed from the Majorbio Bioinformatics Platform (www.majorbio.com) using default parameters. 

### 2.5. Phylogenetic Analysis

The amino acid sequences of UGTs or MYB TFs were aligned using ClustalW program (http://www.ebi.ac.uk/clustalW/). A phylogenetic tree was constructed using the neighbor-joining method using MEGA 6.0 software [26].

### 2.6. Statistical Analysis

Phytochemical data were shown as mean ± SD of three biological replicates. Data analysis was performed through one-way ANOVA. Difference was considered statistically significant when ** *p* < 0.05 and extremely significant when *** *p* < 0.01.

## 3. Results

### 3.1. Differential Accumulation of Puerarin and the Isoflavone Aglycones in the Pueraria Tissues

**Figure 2:**
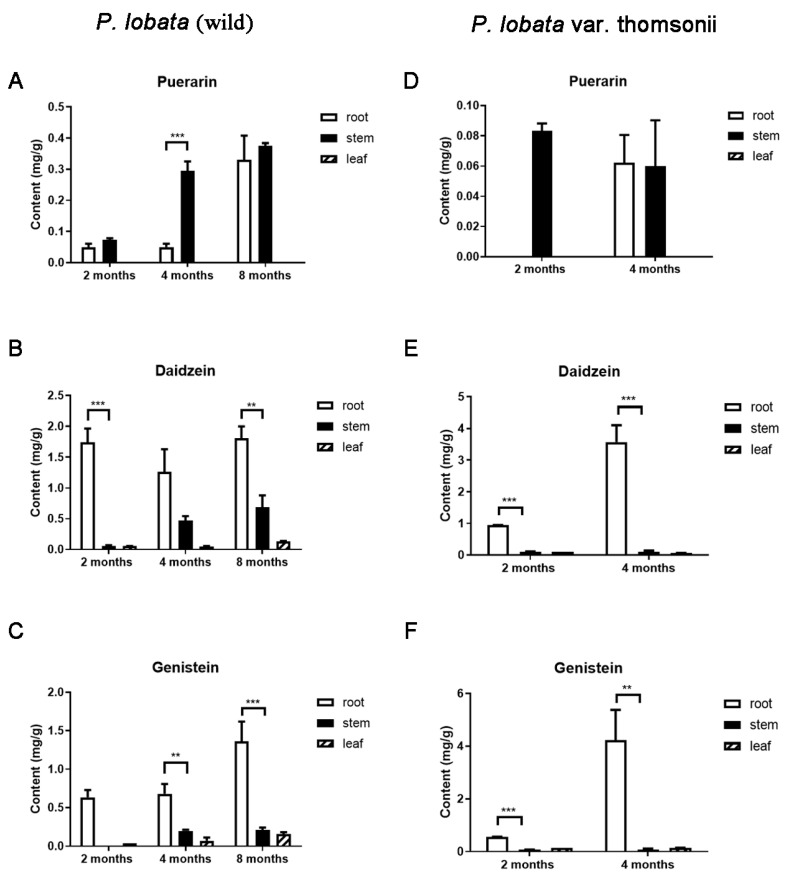
Comparison of the levels of genistein (**C**,**F**), daidzein (**B**,**E**), and puerarin (**A**,**D**) in different tissues at different growth stages of *P. lobata* (wild) and *P. lobata* var. thomsonii. Data are shown as the average with standard errors of three biological replicates at each growth stage. Asterisks indicate statistically significant differences between roots and stems (** *p* < 0.05; *** *p* < 0.01).

We used HPLC to measure the distribution of puerarin in the different tissues of the wild *P. lobata* and its thomsonii cultivar. In addition to puerarin (i.e., daidzein 8-*C*-glucoside), two isoflavone aglycones, daidzein and genistein, were also quantified. To monitor their developmental variations, the *P. lobata* (wild) plants were analyzed at three developmental stages (2, 4, and 8 months after germination), and the thomsonii plants were harvested at two developmental stages (2 and 4 months after germination). In the two *P. lobata* cultivars, puerarin displayed a clearly different tissue-accumulation pattern than that observed for daidzein and genistein. For example, both daidzein and genistein accumulated to their highest levels in the roots of the two cultivars, while much lower levels of them were found in the stem and leaf tissues (Figure 2B,C,E,F). By contrast, puerarin was found in substantially higher amounts, or at least at comparable levels, in the stems compared to the roots over the developmental stages (Figure 2A,D).

Previous studies indicated that daidzein serves as a precursor of puerarin [10,13]. Although daidzein and puerarin displayed different tissue specificities as described above, the temporal accumulation pattern over tissue development was similar for the two compounds. For example, in the thomsonii cultivar, the contents of both daidzein and puerarin in the roots markedly increased from the 2-month to 4-month stage, whereas their contents in the stems all seemed to be constant over these stages (Figure 2D–F). In the *P. lobata* (wild) stems, the levels of both compounds increased from the 2-month stage to the 4-month stage and remained steady at the 8-month stage (Figure 2A–C).

### 3.2. Transcriptomic Analysis of Isoflavone Biosynthetic Genes in P. lobata

The roots were previously considered to be a predominant site of puerarin biosynthesis in the *P. lobata* species [10]. However, a phytochemical analysis of the two *P. lobata* cultivars used here showed that, when the 2-month-old thomsonii plants were analyzed, puerarin was detectable only in the stems (Figure 2F) and that, in the 4-month-old wild *P. lobata* plants, it was accumulated 4.8 times higher in the stems than in the roots (Figure 2C). This result suggested that the stems may also be a primary site, where the transcription of the puerarin biosynthetic genes occurs. Therefore, to further provide insight into puerarin biosynthesis, we performed an RNA sequencing analysis on the root and stem tissues of the two *P. lobata* cultivars, which were the same materials used for the phytochemical analysis mentioned above. Three biological replicates per tissue sample at each stage were employed to generate a cDNA library. After removing the adapter and low-quality sequences, clean reads of each sample were obtained with the values of Q20 and Q30 and the GC content as shown in Table S1. Using the HISAT2 software, the clean reads were separately aligned to the recently published genome [18] of the thomsonii cultivar to get the mapped reads, and 83–95% of the sequenced reads could be aligned to the reference genome (Table S2, Supplementary Materials). Using the StringTie software, in a reference-based approach, the mapped reads were then assembled into unigenes. The functional annotation of the transcripts were conducted through a BLAST analysis against several public databases, which included the NR, GO, KEGG, COG, Swiss-Prot, and Pfam databases, and above 99.0% of the total transcripts were successfully annotated (Table S3, Supplementary Materials).

**Figure 3:**
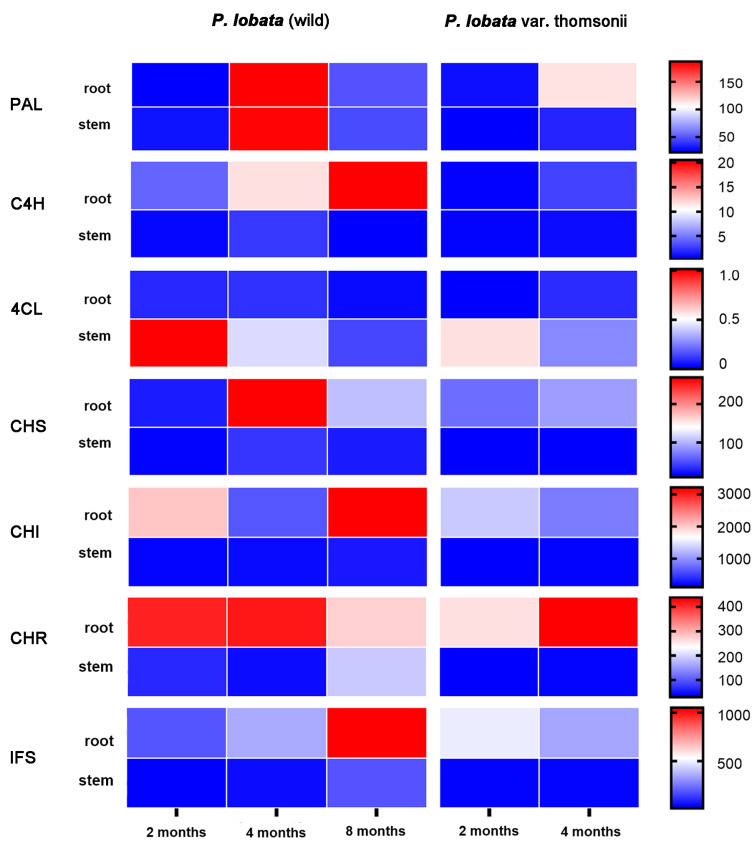
Heatmap representing the expression profile of the upstream isoflavone biosynthetic genes in the analyzed tissues of *P. lobata* (wild) and *P. lobata* var. thomsonii during different stages. The transcript abundances were assessed based on their FPKM values calculated from the RNA-sequencing data.

The analysis of the transcriptome led to the identification of two *PlPALs*, two *PlC4Hs*, three *Pl4CLs*, three *PlCHSs*, two *PlCHIs*, three *PlCHRs*, and one *PlIFS*, which encode the putative enzymes in the biosynthetic steps leading up to daidzein and genistein (Figure 1). In agreement with the finding that daidzein and genistein were predominantly accumulated in the roots of the two *P. lobata* cultivars (Figure 2), all of the upstream isoflavone biosynthetic genes, except for *PAL* and *4CL*, were more strongly expressed in the roots relative to the stems in either cultivar (Table S4, Supplementary Materials). The digital expression patterns of these genes at different stages were also analyzed. Considering that the orthologs of the pathway genes may redundantly and additively contribute to each step of the pathway, we simplified the analysis by summing the FPKM values of the orthologs. Generally, a strong transcript–metabolite correlation was observed in either *P. lobata* cultivar. For example, only a small variation (about 10%) in the levels of the transcripts was observed in the thomsonii stems between the two stages (Figure 3), where consistently a nearly stable accumulation pattern was found for daidzein and genistein (Figure 2). In the thomsonii roots, the expression of these genes markedly increased following the developmental stages (Figure 3), which was also consistent with the increased levels of daidzein and genistein observed in the 4-month-old roots of this plant compared to the 2-month-old roots (Figure 2). In the roots of the wild cultivar, there were four types of expression patterns for these upstream genes across the three stages (i.e., 2, 4, and 8 months after germination) (Figure 3): *C4H* and *IFS* showed a continual increased expression trend during root development; *PAL* and *CHS* showed their highest expression at the 4-month stage, and then their expression decreased at the 8-month stage; *CHI* showed its lowest expression at the 4-month stage; *CHR* was transcribed at a stable level from the 2-month to 4-month stage, and then its expression level almost halved at the 8-month stage. The accumulative effect of the *PAL*, *C4H*, *CHS*, *CHI*, and *IFS* transcripts on metabolite biosynthesis could lead to the accumulation pattern observed for genistein in the *P. lobata* (wild) roots, which was at a relatively stable level at the 2- and 4-month stages and then increased at the 8-month stage (Figure 2). The biosynthesis of daidzein differs from that of genistein by the presence of CHR in the pathway (Figure 1). In view of the expression pattern for *CHR* (Figure 3) and the genistein accumulation profile (Figure 2) in the *P. lobata* (wild) roots, the addition of CHR into the genistein pathway could perfectly explain why daidzein showed a generally stable accumulation pattern at the three stages of the *P. lobata* (wild) roots (Figure 2).

Therefore, the RNA-seq generated gene expression data could spatially and temporally explain the accumulation profiles of the isoflavone aglycones of the two *P. lobata* cultivars.

### 3.3. Identification of Specific UGT Genes Correlated to Puerarin Biosynthesis

**Figure 4:**
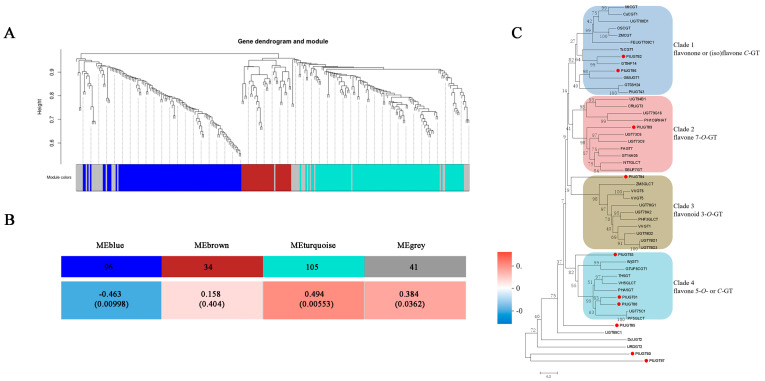
Analysis of the *P. lobata* glycosyltransferase (PlUGT) genes. (**A**) Clustering diagram of the 267 PlUGTs by their gene expression patterns. (**B**) Weighted gene coexpression network analysis (WGCNA) for the correlation of the expression modules consisting of 276 *P. lobata* UGT genes and the puerarin-accumulation trait. (**C**) Phylogenetic analysis of the 10 top-ranked PlUGT candidates selected from this study with the previously published known *C*- and *O*-glucosyltransferases. The tree was constructed using the neighbor-joining method using MEGA 6.0 software with 1000 bootstrap replicates. Accession numbers of the previously characterized *C*- and *O*-UGTs are shown in Table S5 (Supplementary Materials).

The 8-*C*-glucosylation reaction in puerarin biosynthesis is catalyzed by a UDP-glycosyltransferase (UGT). It is not clear whether only one specific UGT is solely involved in this process or if several UGTs are required to synergistically contribute to *C*-glycosylation at the different pathway stages (see Figure 1). In the transcriptome generated by this study, we identified a total of 276 UGTs that showed significant expression levels (a cutoff of FPKM > 1.0 in at least one sample) in the analyzed tissues. In order to identify the UGTs related to puerarin biosynthesis, these 276 *PlUGTs* were subjected to a weighted gene coexpression network analysis (WGCNA), correlating their expression data with the puerarin-accumulation trait. This analysis resulted in four coexpression modules which were named blue (96 PlUGTs), brown (34 PlUGTs), turquoise (105 PlUGTs), and grey (41 PlUGTs) (Figure 4A). From the correlationanalysis of the PlUGT gene module to puerarin-accumulating trait, we identified that the turquoise module was the most highly correlated with puerarin biosynthesis (Figure 4B).

The 10 top-ranked candidates (provisionally termed PlUGT80-PlUGT89) with the highest correlation levels were selected from the turquoise module, and they were subjected to a phylogenetic analysis together with the previously characterized *C*- and *O*-UGTs (see their accession numbers in Table S5, Supplementary Materials). The UGTs in the tree were clustered into four groups following their biochemical functions (Figure 4C). PlUGT80, PlUGT85, and PlUGT87 were not clustered into any groups. PlUGT82 and PlUGT86 were grouped into clade 1, which included several previously characterized flavanone *C*-GTs, including OsCGT [27], UGT708D1 [28], MiCGT [29], UGT708C1 [30], CuCGT1 [31], and ZmCGT [32]; one flavone *C*-GT (TcCGT1) [33]; and one isoflavone *C*-GT (PlUGT43) [10]. In clade 2, PlUGT89 showed a close relationship with the flavone 7-*O*-GTs. PlUGT84 was included in clade 3, where flavonoid 3-*O*-GTs clustered. In clade 4, PlUGT83, PlUGT81, and PlUGT88 were grouped with two flavone *C*-GTs (WjGT1 and GtUF6CGT1) and the flavone 5-*O*-GTs, with PlUGT81 and PlUGT88 being more closely related to the flavone 5-*O*-GTs. Therefore, based on the phylogenetic analysis, we predicted PlUGT82, PlUGT83, and PlUGT86 to be potential plant *C*-GT candidates that may play a role in the 8-*C*-glycosylation reaction in puerarin biosynthesis. The predicted amino acid sequences of PlUGT82, 83, and 86 are shown in Table S6 (Supplementary Materials).

### 3.4. Identification of Specific Transcription Factors Important for Isoflavone Biosynthesis

**Figure 5:**
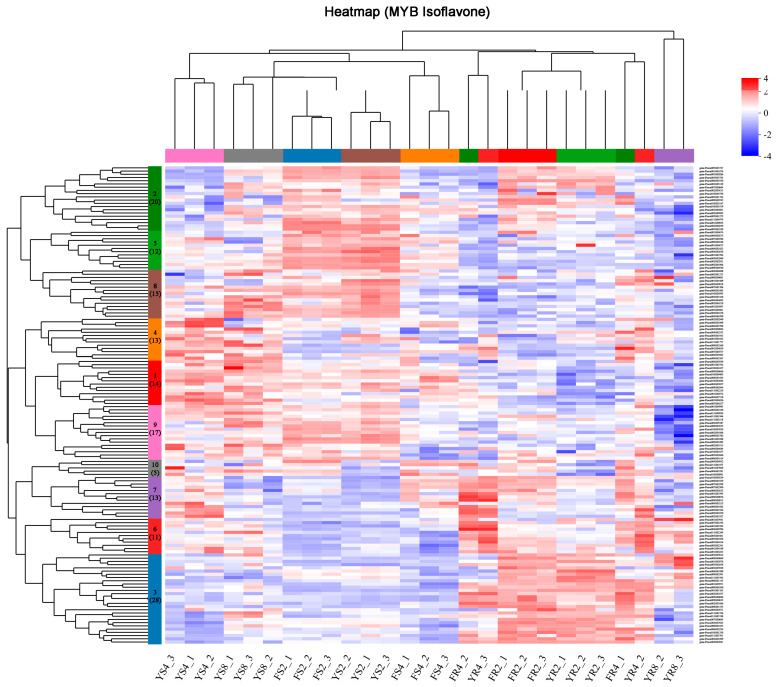
Clustering analysis of the 123 PlMYB sequences according to their expression profiles at the different growth stages of the root and stem tissues of *P. lobata* (wild) and *P. lobata* var. thomsonii. FR represents roots of *P. lobata* var. thomsonii, FS represents stems of *P. lobata* var. thomsonii, YR represents roots of *P. lobata* (wild), and YS represents stems of *P. lobata* (wild). The number next to the sample name indicates the growth stage at which the sample was collected; for example, FR2 represents the 2-month-old roots of *P. lobata* var. thomsonii. The number of PlMYBs in each cluster is indicated in brackets.

Transcription factors (TFs), which specifically bind to cis-acting regulatory elements in the promoter of target genes, can modulate plant secondary metabolite biosynthesis [34]. In the transcriptome generated by this study, a total of 2849 transcripts were annotated as TFs, and they mainly belonged to the *WRKY*, *MYB*, *ERF*, *bHLH*, *C3H*, and *NAC* families. The present study was focused on the identification of the *MYB* genes that may regulate isoflavone biosynthesis in the *P. lobata* species. Initially, 443 sequences, which corresponded to the MYB family TFs, were identified from the transcriptome. After removing the redundant sequences and low-abundance transcripts (a cutoff of FPKM > 1.0 in at least one sample), only 123 *MYB* sequences were retained for further analysis, and they were provisionally named PlMYB1-PlMYB123. The clustering analysis revealed that these 123 *PlMYBs* could be grouped into 10 clusters according to their expression pattern in the tissues of both of the *P. lobata* cultivars during the developmental stages (Figure 5). Through the coexpression analysis, we looked for the *PlMYBs* that showed a positive correlation with the key genes of isoflavone biosynthesis. Based on the expression profile, *PAL* was grouped with the *PlMYBs* in cluster 7, *CHS* and *C4H* were grouped in cluster 6, and *CHI* was grouped in cluster 3.

**Figure 6:**
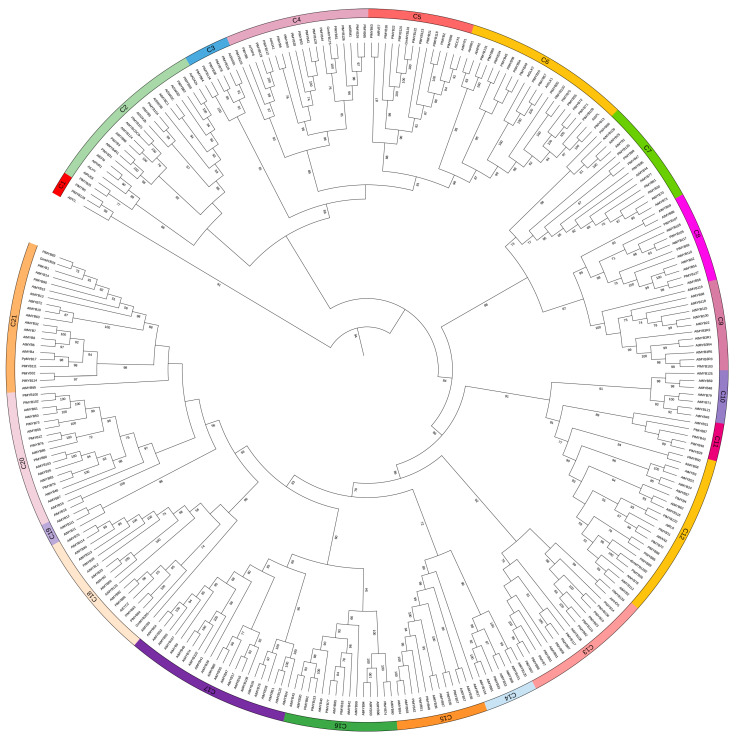
Phylogenetic tree of the 123 *P. lobata* MYBs with the 161 MYB proteins from *Arabidopsis* and 6 MYBs from *Glycine max*. The tree was constructed using the neighbor-joining method using MEGA 6.0 software with 1000 bootstrap replicates. The *Arabidopsis* MYB proteins used for the tree construction of this study were selected based on a previous report [37].

To further evaluate their possible regulatory roles, a phylogenetic analysis was conducted on the 123 PlMYBs together with the previously characterized 161 AtMYBs from *Arabidopsis* and the 6 GmMYBs from *Glycine max*. The GmMYBs were included in the analysis because of their experimentally confirmed roles in regulating isoflavone biosynthesis [16,17]. Based on the phylogenetic tree topology, the *P. lobata* MYB proteins were clustered into 21 clades (C1–C21) (Figure 6). Only three sequences (PlMYB17, PlMYB108, and PlMYB125) were not assigned to any clades (Figure 6). The MYBs in the subfamilies of C4, C5, C6, and C13 were predominantly from the *P. lobata* species (Figure 6), indicating that the PlMYB sequences in these clades experienced expansions after the divergence of *P. lobata* and *Arabidopsis*. Considering that orthologous genes with similar functions are usually clustered in the same clades, the phylogenetic features of the PlMYBs could, therefore, provide insights into their regulatory roles. In clade C5 (Figure 6), PlMYB122 showed the closest relationship to a soybean CCA1-like MYB protein, namely GmMYB133, which is an enzyme that positively regulates the expression of the *IFS* and *CHS* genes for isoflavonoid biosynthesis in soybeans [35]. Similarly, in clade 21, PlMYB83 showed phylogenetic proximity to GmMYB29, which also functions as an activator of *IFS* and *CHS* expression in soybeans [16]. In clade 4, PlMYB61 lay in the same branch as GmMYB176, which is an R1 MYB transcription factor that regulates multiple genes in the isoflavonoid pathway in soybean roots [36]. Therefore, it is worth further investigating whether the three PlMYB candidates (PlMYB61, PlMYB83, and PlMYB122) could regulate puerarin biosynthesis in *P. lobata*. The predicted amino acid sequences of PlMYB61, 83, and 122 are shown in Table S7 (Supplementary Materials).

## 4. Discussion

During the past ten years, extensive attention has been paid to the discovery of tailoring enzymes (e.g., glycosyltransferase and methyltransferase) catalyzing the formation of isoflavones in the *P. lobata* species [10,14,19,38,39,40]. Those efforts heavily depended on a comparative analysis of the transcriptomes of the leaf and root tissues of the *P. lobata* species to target candidates from the differentially expressed genes (DEGs). Dixon et al. [12,13] also carried out a comparative analysis of the transcriptomes generated from two species of the *Pueraria* genus, namely *P. m. lobata* and *P. phaseoloides*, searching for the DEGs that are important for the biosynthesis of isoflavone glycosides, especially for that of puerarin, the characteristic compound in the *P. lobata* species. However, a comparison of the transcripts of the different tissues of the same species or of different *Pueraria* species would lead to a huge number of DEGs from which it would still be difficult to narrow down true candidates for this purpose. Indeed, in spite of some progress being made over the years, the mechanism underlying how puerarin is biosynthesized and how its production is regulated still remains poorly understood. In this study, with the aim of narrowing down the number of candidates, we performed a comparative analysis on the transcriptomes generated from the different developmental stages of the same tissue (root or stem) derived from the two cultivars (i.e., *P. lobata* (wild) and its thomsonii variety) but belonging to the same *P. lobata* species. We confirmed the close relationship of *P. lobata* (wild) with the thomsonii variety by aligning the RNA-sequenced reads of the *P. lobata* (wild) tissues to the genome of the thomsonii variety, which was recently published [18]. As shown in Table S2 (Supplementary Materials), the percentages of the RNA-sequenced reads that were successfully mapped to the thomsonii genome were more than 89% for the *P. lobata* (wild) roots and above 94% for its stems, confirming that both cultivars indeed belong to the same *P. lobata* species. The two *P. lobata* cultivars showed a distinct accumulation pattern for the isoflavones during the different stages (Figure 2), ensuring that they could be valid inputs for a comparative analysis. Either in a spatial or in a temporal manner, the RNA-sequencing-generated expression data could generally match the accumulation patterns for the isoflavone aglycones (such as daidzein and genistein) in the two *P. lobata* cultivars (Figure 2 and Table S4, Supplementary Materials). These observations suggested that the plant materials employed in this study and their corresponding RNA-sequenced data were reliable for further analysis.

Although the upstream metabolic steps leading up to isoflavone aglycone are well characterized, the current understanding of the biosynthesis of isoflavone glycosides (e.g., puerarin) is still somewhat limited. Some data remain in conflict, especially concerning the nature of the precursor of the *C*-glycosyltransferase in puerarin biosynthesis. An early labeling experiment suggested that *C*-glucosylation takes place at the level of chalcone with isoliquiritigenin proposed as a substrate [11]. However, cell-free enzyme assays using *Pueraria* root extracts hinted that the *C*-glucosyl bond of puerarin is formed at the isoflavanone stage using 2-hydroxyisoflavanone as a possible substrate [12]. The latter assumption is prone to being believed because the 2-hydroxyflavanone *C*-GT has been proven to have a role in the biosynthesis of flavone *C*-glycosides in several plant species [27,28,30,32]. However, to the best of our knowledge, the hypothetical 2-hydroxyisoflavanone *C*-GT has not yet been identified in any plant species to date, including those of the *Pueraria* genus. Rather, an isoflavone *C*-GT (namely PlUGT43 or UGT71T5), which mediates the direct *C*-glucosylation of an isoflavone backbone, resulting in puerarin formation, was previously cloned from the *P. lobata* species [10]. A more recent report further convinced such the role of PlUGT43 in puerarin biosynthesis utilizing the *PlUGT43* RNAi-silenced *P. lobata* hairy roots [13]. Questions remain, however, concerning the possibility that another UGT exists, distinct from PlUGT43, that *C*-glucosylates 2-hydroxyisoflavanone during puerarin biosynthesis in *P. lobata*.

In this study, we made continued efforts to search for a potential 2-hydroxyisoflavanone *C*-GT candidate in the *P. lobata* species. In the previous studies [10,12], the gene screening criteria to obtain a shortlist of UGT candidates were focused on the UGT genes which were highly expressed in the *P. lobata* roots that had expression patterns similar to that of *IFS*. However, this study revealed a more pronounced accumulation of puerarin in the stems of two *P. lobata* cultivars compared to their roots (Figure 2). Since secondary metabolites are most often synthesized at or near their site of accumulation [41], we accordingly hypothesized that both the root and stem tissues of *P. lobata* were the sites of puerarin biosynthesis. By RNA-sequencing the different growth stages of the root and stem tissues of the two *P. lobata* cultivars, we generated valuable gene expression data based on the genome of the thomsonii variety for the investigation of puerarin biosynthesis. All the known upstream genes related to the biosynthesis of isoflavones (e.g., daidzein and genistein) could be found in this transcriptome, and, at the different growth stages of the *P. lobata* (wild) roots, they exhibited four distinct types of gene expression profiles with only *C4H* sharing an expression pattern similar to that of the *IFS* gene (Figure 3). Therefore, the strategy of filtrating the UGT candidates according to the *IFS* expression trend, which was previously adopted as the primary criteria [10,12], may not be appropriately suited to targeting the downstream *C*-GT in puerarin biosynthesis. In this study, a total of 276 PlUGTs with significant transcripts in the analyzed tissues of both of the *P. lobata* cultivars were identified, and their gene expression patterns were clustered into 4 modules by WGCNA. The gene module–metabolite trait correlation analysis indicated that the turquoise module comprising 105 PlUGTs was relatively more associated with puerarin biosynthesis (Figure 4A). It should be noted that PlUGT43, the enzyme previously characterized as a daidzein *C*-glucosyltransferase [10], was not placed in this turquoise module. We subjected the top 10 PlUGTs from this module to a phylogenetic analysis. All the selected flavanone *C*-GTs (CuCGT1, MiCGT, UGT708D1, UGT708C1, ZmCGT, and OsCGT1) resulted in a strong clustering in the tree (Figure 4B), indicating that they could be evolutionary descendants of a common ancestor. In contrast, the (iso)flavone *C*-GTs (TcCGT1, WjGT1, GTUF6CGT1, and PlUGT43) were clustered either with the isoflavone 7-*O*-GTs (e.g., GT04F14 and GmUGT1) [12,42] or with the flavone 5-*O*-GTs (Figure 4B), demonstrating that they evolved independently and are not derived from the same ancestral gene. There were no PlUGT candidates that were closely grouped with the flavanone *C*-GTs in the tree; therefore, if there is indeed an isoflavanone *C*-GT that is involved in puerarin biosynthesis in *P. lobata*, it must be distinct from the flavanone *C*-GT. The hypothetical 2-hydroxyisoflavanone *C*-GT may group with the *O*-GTs, as the shift between the *O*- and *C*-GT could take place easily by changing only a few amino acids [33,43]. In this case, although PlUGT82 and PlUGT86 showed the closest relationship with the isoflavone 7-*O*-GTs (e.g., GT04F14 and GmUGT1), they lay within the branch with the flavone *C*-GT and shared a tree trunk with the 2-hydroxyflavanone *C*-GT (Figure 4B). Therefore, PlUGT82 and PlUGT86 could be the priority candidates. PlUGT83 was proposed to be another 2-hydroxyisoflavanone *C*-GT candidate, as it lay within the same clade as WjGT1 and GtUF6CGT1 (Figure 4B), which are flavone *C*-GTs. However, the biochemical function of the UGTs could not be predicted only based on their primary sequences, and whether they play a role in puerarin biosynthesis warrants further investigation.

To date, the regulation of puerarin biosynthesis is not understood. To identify the transcription factors that regulate puerarin biosynthesis, we performed a genome-wide analysis of the MYB sequences in *P. lobata*, identifying 123 nonredundant PlMYBs. The PlMYBs were clustered into ten groups according to their gene expression patterns in the analyzed tissues during the different growth stages, and the PlMYB members of clusters 3, 6, and 7 generally showed higher expression levels in the roots than in the stems (Figure 5). Interestingly, the gene coexpression analysis revealed that the PlMYBs showing a positive correlation with the isoflavone biosynthetic genes were included only in clusters 3, 6, and 7 (Figure 5), indicating their roles in regulating isoflavone biosynthesis in *P. lobata*. An extensive phylogenetic analysis of the PlMYBs with the known MYB members of *Arabidopsis* and *Glycine max* revealed three candidates (PlMYB61, 83, and 122) that may play a regulatory role in isoflavone biosynthesis. It should be noted that PlMYB83 was included in cluster 6 as mentioned above, thereby allowing us to propose that PlMYB83 could be the best candidate worthy of being selected for further experiments. Unlike the three *Arabidopsis* MYB members (AtMYB11, AtMYB12, and AtMYB111) that fell into the same clade (clade 19) of the tree, the soybean MYBs (GmMYB29, 12B2, 133, 176, and 205) related to isoflavonoid biosynthesis were scattered throughout different clades (Figure 6), which implied that, in the legume species, the MYB proteins related to isoflavonoid biosynthesis were not evolutionarily conserved.

## 5. Conclusions

Phytochemical studies of the different tissues of the two *P. lobata* varieties proposed that both the roots and stems are the biosynthetic sites of puerarin. RNA sequencing of the root and stem tissues during the different developmental stages was performed using an Illumina NovaSeq6000 sequencer, and a *P. lobata* transcriptome was reconstructed based on the recently published genome of *P. lobata* var. thomsonii. A gene coexpression network analysis combined with metabolite profiling identified the gene module correlated with puerarin biosynthesis from which a further phylogeny analysis revealed several *C*-GT candidates that may participate in puerarin biosynthesis. A genome-wide analysis of the whole MYB superfamily revealed the entire complement of the MYBs in *P. lobata*. The detailed phylogenetic analysis proposed strong PlMYB candidates that may regulate the biosynthesis of isoflavonoids in *P. lobata*.

## Data Availability

The original contributions presented in this study are included in this article/in the Supplementary Materials, and further inquiries can be directed to the corresponding author/s.

## Supplementary Materials

The following supporting information can be downloaded at https://www.mdpi.com/article/10.3390/biom13010170/s1, Table S1: Summary of the RNA-sequencing data; Table S2: Summary statistics of the mapping of RNA-sequencing reads derived from each sample of this study to the genome of *P. lobata* var. thomsonii; Table S3: Summary statistics of functional annotations for unigenes and transcripts of *P. lobata* via public databases; Table S4: Transcript abundances of upstream genes for the biosynthesis of isoflavones in each *P. lobata* sample; Table S5: Accession number of the previously characterized C- and O-UGTs selected for the phylogenetic analysis in this study; Table S6: Predicted amino acid sequences of the three PlUGT candidates in this study; Table S7: Predicted amino acid sequences of the three PlMYB candidates in this study.

## References

[1] Wang S., Zhang S., Wang S., Gao P., Dai L. (2020). A comprehensive review on *Pueraria*: Insights on its chemistry and medicinal value. Biomed. Pharmacother..

[2] Wong K.H., Li G.Q., Li K.M., Razmovski-Naumovski V., Chan K. (2011). Kudzu root: Traditional uses and potential medicinal benefits in diabetes and cardiovascular diseases. J. Ethnopharmacol..

[3] Zhou Y.X., Zhang H., Peng C. (2014). Puerarin: A review of pharmacological effects. Phytother. Res..

[4] Zhou Y.X., Zhang H., Peng C. (2021). Effects of Puerarin on the Prevention and Treatment of Cardiovascular Diseases. Front. Pharmacol..

[5] Kulczynski B., Gramza-Michalowska A., Suliburska J., Sidor A. (2021). Puerarin-an isoflavone with beneficial effects on bone health. Front. Biosci..

[6] Murahari M., Singh V., Chaubey P., Suvarna V. (2020). A Critical Review on Anticancer Mechanisms of Natural Flavonoid Puerarin. Anti-Cancer Agents Med. Chem..

[7] Wang H.X., Zeng M.S., Ye Y., Liu J.Y., Xu P.P. (2021). Antiviral activity of puerarin as potent inhibitor of influenza virus neuraminidase. Phytother. Res..

[8] Jung W., Yu O., Lau S.M., O’Keefe D.P., Odell J., Fader G., McGonigle B. (2000). Identification and expression of isoflavone synthase, the key enzyme for biosynthesis of isoflavones in legumes. Nat. Biotechnol..

[9] Vogt T. (2010). Phenylpropanoid biosynthesis. Mol. Plant.

[10] Wang X., Li C., Zhou C., Li J., Zhang Y. (2017). Molecular characterization of the C-glucosylation for puerarin biosynthesis in Pueraria lobata. Plant J. Cell Mol. Biol..

[11] Inoue T., Fujita M. (1977). Biosynthesis of puerarin in Pueraria root. Chem. Pharm. Bull..

[12] He X., Blount J.W., Ge S., Tang Y., Dixon R.A. (2011). A genomic approach to isoflavone biosynthesis in kudzu (*Pueraria lobata*). Planta.

[13] Adolfo L.M., Burks D., Rao X., Alvarez-Hernandez A., Dixon R.A. (2022). Evaluation of pathways to the C-glycosyl isoflavone puerarin in roots of kudzu (*Pueraria montana lobata*). Plant Direct.

[14] Li J., Li Z., Li C., Gou J., Zhang Y. (2014). Molecular cloning and characterization of an isoflavone 7-O-glucosyltransferase from *Pueraria lobata*. Plant Cell Rep..

[15] Prasain J.K., Reppert A., Jones K., Moore D.R., Barnes S., Lila M.A. (2007). Identification of isoflavone glycosides in *Pueraria lobata* cultures by tandem mass spectrometry. Phytochem. Anal..

[16] Chu S., Wang J., Zhu Y., Liu S., Zhou X., Zhang H., Wang C.E., Yang W., Tian Z., Cheng H. (2017). An R2R3-type MYB transcription factor, GmMYB29, regulates isoflavone biosynthesis in soybean. PLoS Genet..

[17] Han X., Yin Q., Liu J., Jiang W., Di S., Pang Y. (2017). *GmMYB58* and *GmMYB205* are seed-specific activators for isoflavonoid biosynthesis in Glycine max. Plant Cell Rep..

[18] Shang X., Yi X., Xiao L., Zhang Y., Huang D., Xia Z., Ou K., Ming R., Zeng W., Wu D. (2022). Chromosomal-level genome and multi-omics dataset of *Pueraria lobata* var. *thomsonii* provide new insights into legume family and the isoflavone and puerarin biosynthesis pathways. Hortic. Res..

[19] Wang X., Li S., Li J., Li C., Zhang Y. (2015). De novo transcriptome sequencing in *Pueraria lobata* to identify putative genes involved in isoflavones biosynthesis. Plant Cell Rep..

[20] Cerveau N., Jackson D.J. (2016). Combining independent de novo assemblies optimizes the coding transcriptome for nonconventional model eukaryotic organisms. BMC Bioinform..

[21] Chen S., Zhou Y., Chen Y., Gu J. (2018). Fastp: An ultra-fast all-in-one FASTQ preprocessor. Bioinformatics.

[22] Kim D., Langmead B., Salzberg S.L. (2015). HISAT: A fast spliced aligner with low memory requirements. Nat. Methods.

[23] Pertea M., Pertea G.M., Antonescu C.M., Chang T.C., Mendell J.T., Salzberg S.L. (2015). StringTie enables improved reconstruction of a transcriptome from RNA-seq reads. Nat. Biotechnol..

[24] Mortazavi A., Williams B.A., McCue K., Schaeffer L., Wold B. (2008). Mapping and quantifying mammalian transcriptomes by RNA-Seq. Nat. Methods.

[25] Langfelder P., Horvath S. (2008). WGCNA: An R package for weighted correlation network analysis. BMC Bioinform..

[26] Tamura K., Stecher G., Peterson D., Filipski A., Kumar S. (2013). MEGA6: Molecular Evolutionary Genetics Analysis version 6.0. Mol. Biol. Evol..

[27] Brazier-Hicks M., Evans K.M., Gershater M.C., Puschmann H., Steel P.G., Edwards R. (2009). The C-glycosylation of flavonoids in cereals. J. Biol. Chem..

[28] Hirade Y., Kotoku N., Terasaka K., Saijo-Hamano Y., Fukumoto A., Mizukami H. (2015). Identification and functional analysis of 2-hydroxyflavanone C-glucosyltransferase in soybean (*Glycine max*). FEBS Lett..

[29] Chen D., Chen R., Wang R., Li J., Xie K., Bian C., Sun L., Zhang X., Liu J., Yang L. (2015). Probing the Catalytic Promiscuity of a Regio- and Stereospecific C-Glycosyltransferase from Mangifera indica. Angew. Chem..

[30] Nagatomo Y., Usui S., Ito T., Kato A., Shimosaka M., Taguchi G. (2014). Purification, molecular cloning and functional characterization of flavonoid C-glucosyltransferases from *Fagopyrum esculentum* M. (buckwheat) cotyledon. Plant J. Cell Mol. Biol..

[31] Ito T., Fujimoto S., Suito F., Shimosaka M., Taguchi G. (2017). C-Glycosyltransferases catalyzing the formation of di-C-glucosyl flavonoids in citrus plants. Plant J. Cell Mol. Biol..

[32] Falcone Ferreyra M.L., Rodriguez E., Casas M.I., Labadie G., Grotewold E., Casati P. (2013). Identification of a bifunctional maize C- and O-glucosyltransferase. J. Biol. Chem..

[33] He J.B., Zhao P., Hu Z.M., Liu S., Kuang Y., Zhang M., Li B., Yun C.H., Qiao X., Ye M. (2019). Molecular and Structural Characterization of a Promiscuous C-Glycosyltransferase from *Trollius chinensis*. Angew. Chem..

[34] Vom Endt D., Kijne J.W., Memelink J. (2002). Transcription factors controlling plant secondary metabolism: What regulates the regulators?. Phytochemistry.

[35] Bian S., Li R., Xia S., Liu Y., Jin D., Xie X., Dhaubhadel S., Zhai L., Wang J., Li X. (2018). Soybean CCA1-like MYB transcription factor GmMYB133 modulates isoflavonoid biosynthesis. Biochem. Biophys. Res. Commun..

[36] Anguraj Vadivel A.K., McDowell T., Renaud J.B., Dhaubhadel S. (2021). A combinatorial action of GmMYB176 and GmbZIP5 controls isoflavonoid biosynthesis in soybean (*Glycine max*). Commun. Biol..

[37] Arce-Rodriguez M.L., Martinez O., Ochoa-Alejo N. (2021). Genome-Wide Identification and Analysis of the MYB Transcription Factor Gene Family in Chili Pepper (*Capsicum* spp.). Int. J. Mol. Sci..

[38] Wang X., Li C., Zhou Z., Zhang Y. (2019). Identification of Three (Iso)flavonoid Glucosyltransferases From *Pueraria lobata*. Front. Plant Sci..

[39] Li J., Li C., Gou J., Wang X., Fan R., Zhang Y. (2016). An Alternative Pathway for Formononetin Biosynthesis in *Pueraria lobata*. Front. Plant Sci..

[40] Li J., Li C., Gou J., Zhang Y. (2016). Molecular Cloning and Functional Characterization of a Novel Isoflavone 3′-O-methyltransferase from *Pueraria lobata*. Front. Plant Sci..

[41] Zhang Y., Teoh K.H., Reed D.W., Maes L., Goossens A., Olson D.J., Ross A.R., Covello P.S. (2008). The molecular cloning of artemisinic aldehyde Delta11(13) reductase and its role in glandular trichome-dependent biosynthesis of artemisinin in *Artemisia annua*. J. Biol. Chem..

[42] Funaki A., Waki T., Noguchi A., Kawai Y., Yamashita S., Takahashi S., Nakayama T. (2015). Identification of a Highly Specific Isoflavone 7-O-glucosyltransferase in the soybean (*Glycine max* (L.) Merr.). Plant Cell Physiol..

[43] Gutmann A., Nidetzky B. (2012). Switching between O- and C-Glycosyltransferase through Exchange of Active-Site Motifs. Angew. Chem..

