# Supplementary material for: Comparative Transcriptome Analysis of Pueraria lobata Provides Candidate Genes Involved in Puerarin Biosynthesis and Its Regulation
Source: Biomolecules. 2023 Jan 13;13(1):170. doi: 10.3390/biom13010170 (PMC9855344; doi:10.3390/biom13010170)
Supplement: Supplementary file 1 [file biomolecules-13-00170-s001.zip › biomolecules-2113514-supplementary.pdf]

Supplementary Material

Comparative Transcriptome Analysis of *Pueraria lobata* Provides Candidate Genes Involved in Puerarin Biosynthesis and Its Regulation

Huiting Xi [1], Yaru Zhu [1,2], Wenwen Sun [1], Nan Tang [1], Zhengqin Xu [1], Xiaohong Shang [3], Yansheng Zhang [1], Huabing Yan [3,*] and Changfu Li [1,*]

[1] Shanghai Key Laboratory of Bio-Energy Crops, Research Center for Natural Products, Plant Science Center, School of Life Sciences, Shanghai University, Shanghai 200444, China
[2] Key Laboratory of Plant Germplasm Enhancement and Specialty Agriculture, Wuhan Botanical Garden, Chinese Academy of Sciences, Wuhan 430074, China
[3] Cash Crops Research Institute, Guangxi Academy of Agricultural Sciences, Nanning 530007, China
* Correspondence: changfuli@shu.edu.cn (C.L.); h.b.yan@hotmail.com (H.Y.)

Supplemental Tables

Supplementary Table S1. Summary of the RNA-sequencing data.

Sample	Raw reads	Raw bases	Clean reads	Clean bases	Error rate (%)	Q20 (%)	Q30 (%)	GC content (%)
YR2_1	51162820	7725585820	44856544	6621261977	0.0278	96.9	91.75	47.44
YR2_2	53167924	8028356524	45647684	6755767590	0.0281	96.76	91.46	47.79
YR2_3	53813846	8125890746	44872390	6612975199	0.0287	96.58	91.04	47.61
YR4_1	44944130	6786563630	42900870	6387950619	0.028	96.79	91.5	46.43
YR4_2	44628816	6738951216	42525438	6311530613	0.0287	96.56	90.92	46.57
YR4_3	49413614	7461455714	46226424	6789406799	0.0278	96.92	91.71	46.53
YR8_1	47704494	7203378594	40800462	6017310596	0.0281	96.78	91.52	47.12
YR8_2	58092372	8771948172	45638182	6718943388	0.0283	96.66	91.34	48.26
YR8_3	50491314	7624188414	44951144	6632882940	0.0279	96.83	91.71	48.86
YS2_1	44305888	6690189088	42802992	6385801024	0.0281	96.82	91.43	46.35
YS2_2	44737786	6755405686	43041360	6419808839	0.0275	97.03	91.96	47.25
YS2_3	44808520	6766086520	43011638	6420113402	0.0276	96.98	91.87	46.64
YS4_1	44809584	6766247184	43004166	6414884544	0.0283	96.72	91.25	46
YS4_2	44581388	6731789588	42784410	6406097475	0.0279	96.87	91.59	45.93
YS4_3	44961618	6789204318	43057166	6426743109	0.0281	96.8	91.45	46.21
YS8_1	41972032	6337776832	40473576	6035407369	0.0278	96.91	91.63	46.12
YS8_2	42444138	6409064838	40867750	6095473100	0.0282	96.76	91.31	47.03
YS8_3	45061596	6804300996	43294590	6441813760	0.0279	96.86	91.6	46.62
FR2_1	52058780	7860875780	43481058	6433916382	0.0277	96.96	91.83	47.59
FR2_2	47865334	7227665434	43171002	6403156513	0.0278	96.89	91.72	46.71
FR2_3	49266072	7439176872	45334738	6702661418	0.0287	96.55	90.99	46.69

FR4_1	44713950	6751806450	42900324	6390412104	0.0281	96.81	91.48	46.3
FR4_2	44718466	6752488366	42990216	6410388734	0.0285	96.64	91.11	46.25
FR4_3	44619468	6737539668	42742730	6369138655	0.0278	96.88	91.68	48.09
FS2_1	44103664	6659653264	42665694	6367180846	0.0276	97	91.87	47.36
FS2_2	42530190	6422058690	41093576	6153510720	0.028	96.84	91.43	47.85
FS2_3	44468644	6714765244	42763658	6383896502	0.0278	96.91	91.67	47.38
FS4_1	45082096	6807396496	43223912	6447150247	0.0279	96.86	91.57	47.06
FS4_2	44709040	6751065040	43172778	6449782035	0.0271	97.2	92.31	47.72
FS4_3	43062796	6502482196	41529684	6189382268	0.0276	97	91.88	47.71

Note: YR and YS represent the root and stem of *P.lobata* (wild); FR and FS represent the root and stem of *P.lobata* var. thomsonii; The number next to the sample name refers to the stage at which the sample was harvested; for example, YR2 represents the 2 month-old root of *P.lobata* (wild); Three biological replicates were performed for each stage.

Supplementary Table S2. Summary statistics of the mapping of RNA-sequencing reads derived from each sample of this study to the genome of *P.lobata* var.thomsonii.

Sample	Total reads	Total mapped	Multiple mapped	Uniquely mapped
YR2_1	44856544	40620614(90.56%)	8051499(17.95%)	32569115(72.61%)
YR2_2	45647684	40983890(89.78%)	8115435(17.78%)	32868455(72.0%)
YR2_3	44872390	40885316(91.11%)	8147883(18.16%)	32737433(72.96%)
YR4_1	42900870	39660543(92.45%)	8530594(19.88%)	31129949(72.56%)
YR4_2	42525438	38754116(91.13%)	8030927(18.88%)	30723189(72.25%)
YR4_3	46226424	42245999(91.39%)	8700034(18.82%)	33545965(72.57%)
YR8_1	40800462	36588924(89.68%)	6862925(16.82%)	29725999(72.86%)
YR8_2	45638182	40935009(89.69%)	7697446(16.87%)	33237563(72.83%)
YR8_3	44951144	37333448(83.05%)	7038350(15.66%)	30295098(67.4%)
YS2_1	42802992	40422203(94.44%)	9061214(21.17%)	31360989(73.27%)
YS2_2	43041360	40736914(94.65%)	12180374(28.3%)	28556540(66.35%)

YS2_3	43011638	40576729(94.34%)	10090537(23.46%)	30486192(70.88%)
YS4_1	43004166	40189599(93.46%)	7735296(17.99%)	32454303(75.47%)
YS4_2	42784410	40335702(94.28%)	8214082(19.2%)	32121620(75.08%)
YS4_3	43057166	40318256(93.64%)	8496949(19.73%)	31821307(73.9%)
YS8_1	40473576	38098492(94.13%)	8963433(22.15%)	29135059(71.99%)
YS8_2	40867750	38398361(93.96%)	10461225(25.6%)	27937136(68.36%)
YS8_3	43294590	40690288(93.98%)	9108043(21.04%)	31582245(72.95%)
FR2_1	43481058	37817487(86.97%)	7020851(16.15%)	30796636(70.83%)
FR2_2	43171002	40137879(92.97%)	7293566(16.89%)	32844313(76.08%)
FR2_3	45334738	41834367(92.28%)	7630910(16.83%)	34203457(75.45%)
FR4_1	42900324	40112987(93.5%)	8346692(19.46%)	31766295(74.05%)
FR4_2	42990216	39820627(92.63%)	7826272(18.2%)	31994355(74.42%)
FR4_3	42742730	39690545(92.86%)	7846304(18.36%)	31844241(74.5%)
FS2_1	42665694	40488706(94.9%)	11160843(26.16%)	29327863(68.74%)
FS2_2	41093576	38736581(94.26%)	12140995(29.54%)	26595586(64.72%)
FS2_3	42763658	40488533(94.68%)	11869774(27.76%)	28618759(66.92%)
FS4_1	43223912	40815386(94.43%)	11107901(25.7%)	29707485(68.73%)
FS4_2	43172778	40820604(94.55%)	13147681(30.45%)	27672923(64.1%)
FS4_3	41529684	39220312(94.44%)	12747353(30.69%)	26472959(63.74%)

Note: Multiple mapped reads mean that the reads were mapped to multiple locations of the genome, and uniquely mapped reads represent the reads specifically mapped to a single location of the genome; the mapped percentage is shown in brackets.

Supplementary Table S3. Summary statistics of functional annotations for unigenes and transcripts from *P.lobata* via public databases.

	Expre_Gene number（percent）	Expre_Transcript number（percent）	All_Gene number（percent）	All_Transcript number（percent）
GO	44931(0.8776)	44293(0.8773)	48360(0.8779)	48360(0.8779)
KEGG	21669(0.4232)	21331(0.4225)	23007(0.4176)	23007(0.4176)
COG	49182(0.9606)	48510(0.9609)	52499(0.953)	52499(0.953)
NR	50999(0.9961)	50285(0.996)	54881(0.9962)	54881(0.9962)
Swiss-Prot	44189(0.8631)	43566(0.8629)	47376(0.86)	47376(0.86)
Pfam	40246(0.7861)	39627(0.7849)	43392(0.7877)	43392(0.7877)
Total_anno	51006(0.9962)	50292(0.9962)	54888(0.9964)	54888(0.9964)
Total	51199(1.0)	50485(1.0)	55088(1)	55088(1)

Note: Expre_Gene number represents the number of genes whose fpkm value is above 0 in at least one sample sequenced; Expre_Transcript number represents the number of transcripts whose fpkm value is above 0 in at least one sample; All_Gene number means the number of genes in total; All_Transcript number means the number of transcripts in total; The percentage of genes or transcripts having successfully been annotated via each public database is given in brackets.

Supplementary Table S4. Transcript abundances of upstream genes for the biosynthesis of isoflavones in each *P.lobata* sample.

	YR2	YR4	YR8	YS2	YS4	YS8	FR2	FR4	FS2	FS4
PlPAL-1	16.89667	111.5	15.85	15.62667	57.49333	16.26	20.10333	74.72	13.26667	18.26333
PlPAL-2	5.04	74.36667	32.45333	14.34	124.2133	29.48333	9.026667	38.57333	9.286667	16.62
PlC4H-1	4.126667	11.57333	20.49	0.626667	2.48	0.14	0.3	2.85	0.44	0.9
PlC4H-2	0	2.73	4.003333	0	0.393333	0.016667	0	0.346667	0	0.043333
Pl4CL-1	0	0	0	0.5	0.126667	0.006667	0	0.026667	0.446667	0.24
Pl4CL-2	0.086667	0.103333	0.006667	0	0	0.13	0	0.04	0	0
Pl4CL-3	0.25	0	0	0.03	0.513333	0.306667	0.006667	0	0.026667	0.123333
PlCHS-1	15.97	104.27	44.48	5.14	16.03667	8.336667	31.52	34.83667	5.346667	3.473333

PlCHS-2	1.993333	14.67667	10.22	2.15	2.783333	3.696667	0.97	14.51667	0.196667	1.29
PlCHS-3	8.976667	149.8867	51.44	5.596667	19.47	14.11333	32.37	39.29333	4.846667	4.976667
PlCHI-1	1825.443	434.0967	3037.16	115.6167	177.6167	215.38	1138.3	623.1267	77.13667	129.9767
PlCHI-2	192.9	210.09	175.96	68.07	47.83333	78.12	188.8067	231.67	35.31333	31.39667
PlCHR-1	357.0067	374.74	261.69	42.52	38.08	96.11667	192.5433	396.85	20.15333	22.57333
PlCHR-2	46.90667	36.51667	10.71	20.36	5.383333	86.87	60.78333	35.27333	9	9.153333
PlCHR-3	4.413333	4.21	0.953333	1.71	1.42	6.923333	6.823333	7.713333	0.366667	5.523333
PlIFS	183.39	352.2233	1049.823	8.973333	47.92	180.67	487.7967	343.2867	21.39	29.85

Note: The transcript abundance is represented by the FPKM values retrieved from the RNA-sequencing data, and each value is the average of three biological replicates.

Supplementary Table S5. Accession number of the previously characterized *C*- and *O*-UGTs selected for the phylogenetic analysis of this study.

Name	GenBank accession number	Biochemical property	Host plant species
MiCGT	A0A0M4KE44	Flavanone *C*- glucosyltransferase	*Mangifera indica*
CuCGT1	A0A224AKZ9	Flavanone *C*- glucosyltransferase	*Citrus unshiu*
TcCGT1	QCZ42162	Flavone *C*- glucosyltransferase	*Trollius chinensis*
DcUGT2	A0A291PQH4	Carminate *C*- glucosyltransferase	*Dactylopius coccus*
WjGT1	BBI55602	Flavone *C*- glucosyltransferase	*Eutrema japonicum*
OsCGT	Q5VMI0	Flavanone *C*- glucosyltransferase	*Oryza sativa*
UGT78D1	NP_564357	Flavonol 3-*O*- glucosyltransferase	*Arabidopsis thaliana*
UGT78D2	NP_197207	Flavonol 3-*O*- glucosyltransferase	*Arabidopsis thaliana*
UGT78D3	NP_197205	Flavonol 3-*O*-glucosyltransferase	*Arabidopsis thaliana*
UGT89C1	Q9LNE6	Flavonol 7-*O*-rhamnosyltransferase	*Arabidopsis thaliana*
UGT73C6	NP_181217	Flavone 7-*O*-glycosyltransferase	*Arabidopsis thaliana*

UGT75C1	NP_001348274	Flavone 5-*O*-glycosyltransferase	*Solanum lycopersicum*
UGT78A2	BAD06514	Anthocyanin 3-*O*-galactosyltransferase	*Aralia cordata*
UGT94B1	Q5NTH0	Cyanidin-3-*O*-glucoside 2-*O*-glucuronosyltransferase	*Bellis perennis*
CRUGT3	KAI5656423	Flavone 7-*O*-glycosyltransferase	*Catharanthus roseus*
FEUGT708C1	BAP90360	Flavanone *C*- glucosyltransferase	*Fagopyrum esculentum*
FAGT7	Q2V6J9	Flavone 3-*O*-glucosyltransferase	*Fragaria x ananassa*
GtUF6CGT1	A0A0B6VIJ5	Flavone *C*-glucosyltransferase	*Gentiana triflora*
UGT708D1	NP_001347241	Flavanone *C*-glucosyltransferase	*Glycine max*
GMUGT1	NP_001279020	Isoflavone 7-*O*-glucosyltransferase	*Glycine max*
UGT79G16	Q53UH5	Anthocyanidin 3-*O*-glucoside 2''-*O*-glucosyltransferase	*Ipomoea purpurea*
UGT73C8	XP_003625716	Flavone 7-*O*-glycosyltransferase	*Medicago truncatula*
UGT78G1	A6XNC6	Flavonoid 3-*O*-glucosyltransferase	*Medicago truncatula*
NT7GLCT	Q9AT54	Flavone 7-*O*-glycosyltransferase	*Nicotiana tabacum*
PHF3GLCT	Q9SBQ8	Flavonol 3-*O*-glucosyltransferase	*Petunia x hybrida*
PH1–6RHAT	CAA50376	Anthocyanin 3 glucoside: rhamnosyltransferase	*Petunia x hybrida*
PF5GLCT	NP_193146	Flavone 5-*O*-glucosyltransferase	*Arabidopsis thaliana*
PHA5GT	BAA89009	Anthocyanin 5-*O*-glucosyltransferase	*Petunia x hybrida*
GT04F14	ADV71364	Isoflavone 7-*O*-glucosyltransferase	*Pueraria lobata*
GT14A05	ADV71369	Flavone 7-*O*-glucosyltransferase	*Pueraria lobata*
GT03H24	ADV71363	N/A	*Pueraria lobata*
SBUF7GT	BAA83484	Flavonoid 7-*O*-glucosyltransferase	*Scutellaria baicalensis*
URDGT2	AAF00209	Antibiotic urdamycin *C*-glucosyltransferase	*Streptomyces fradiae*
TH5GT	BAC54093	Anthocyanidin 5-*O*-glucosyltransferase	*Torenia hybrid cultivar*
VVGT6	NP_001267832	Anthocyanidin 3-*O*-glucosyltransferase	*Vitis vinifera*

VH5GLCT	Q9ZR25	Anthocyanidin 3-*O*-glucoside 5-*O*-glucosyltransferase	*Glandularia x hybrida*
VVGT1	NP_001384786	Anthocyanidin 3-*O*-glucosyltransferase	*Vitis vinifera*
VVGT5	BAI22846	Anthocyanidin 5-*O*-glucosyltransferase	*Vitis vinifera*
ZMCGT	NP_001132650	Flavanone *C*-glucosyltransferase	*Zea mays*
ZM3GLCT	P16165	Anthocyanidin 3-*O*-glucosyltransferase	*Zea mays*
PlUGT43	A0A172J2G3	Isoflavone *C*-glucosyltransferase	*Pueraria lobata*

Supplementary Table S6. The predicted amino acid sequences of the three PlUGT candidates of this study.

PlUGT candidates	Predicted amino acid sequences
PlUGT82	mgktthiavipsagfshfapilqfskrlvelhpdfhvtcivpiigslpssskailqtlppninpvflppvnlddqpqgvpvvvqiqlamsham<br>psihhtlksitsstphaamvvdsfasealdfahelnmlsyvyfpcsattlsthfylpkldeetsceyrdlpdpievpgcvpfhgrdlysqaqdrs<br>sqlykvslkrykryrsvdgiimnsflaletgpiralkdeergyphvypigpivqtgtdsakglewltwldkqqvgsvlyvsfgsggtlsqeq<br>mnelayglelsnhkflwvvrapnnadnsaylgeqivdplqflpsgflertkeqgvvipswapqvqilshssvggflthcgwnstletvlhgv<br>pvitwplyaeqrmnavllceglkvgvrprvsenglvekeeiaevikclmegeegakmrkrmkeleeaatnalkeggsstetlselalmwk<br>gla
PlUGT83	mvqqrqnnvhvivlpypaqghinplvqfakrlaskgvkatvatthytansiaapnitveaisdgfdeagfaqtnnnvqlfltsfrtngsrtlsql<br>iqkhqqtpspvtcivydsffpwaldvakqhgiygaafftnsaavcnifcrihhgfielpvkmedlplrvpglppldsralpsfvkfpqsypay<br>mamklsqfsnlneadwmfvntfealegevvkglteffpakmigpmvpsgyldgrikgdkgygaslwkplteecsnwleakapqsvvy<br>isfgsmvsltaeqmeevawglkesgvsflwvlresehgklpsgyrdlvkdkglivtwcnqlellahqatgcfvthcgwnstleslslgvpvv<br>clpqwadqlpdakfldeiwevgvwpkedekgiirkqefvkslkvvmegersqeirrnankwkklareavgeggssdkhinqfvdhlm<br>nadkngslnvy
PlUGT86	meqqdtntivlypapgighivsmvelakllqthhysitillttgfldhpsidayihristshpsisfhrlphtalaatpttvsraakafdfikrntpn<br>vsttltrisksttikafvidlfctsamesasslgipvyyfftsgaavlalfsyfpklheetsmsfkdmvgvelrvpgnaplravnmpepvlerdd<br>paywdmlyfcthlpkargiivnsfpelepvavkaiadgacfpnpehappvyymgpliaepqpqqpdvatdskqclswldaqasrsvvfl<br>cfgslgsfpvsqlreianglersghrflwvvkrpiqdegtklihdtttgefdlssvlpsgfiertkdrgivvrtwapqvevlsrdsvgafvshcg<br>wnsvlegvvagvpmvawplyaeqhvnrhvmvgemkvavaveqreedgfvsgeevekrvrevmeskevretsfklkqlalaavgesg<br>ssttalanlvhswv

Supplementary Table S7. The predicted amino acid sequences of the three PlMYB candidates of this study.

PlMYB candidates	Predicted amino acid sequences
PlMYB61	msracaaadsaasgeimlfgvrvvvdsmrksvsmnnlsqyehpqdannnnnkdslaagyasaddaaphnsgrhrererkrgvpwtee<br>ehklflvglqkvgkgdwrgisknyvktrtptqvashaqkyflrrsnlnrrrrrsslfdittdtvsaipieeeqvqnqdtllshsqpvcpavpetsk<br>isgfpmmpvyqfgvgsgvisvqggnpmeeltlgqgnvekhnvpnkastvsdiiapssssavdpptlslglsfssdqrqtssrhsalhaiqcf<br>sngdsiisva
PlMYB83	mglkkgpwapeedqiitsyiekhghgnwralpkqagllrcgkscrlrwinylrpdikrgnftieeeetiiklhemlgnrwsaiaaklpgrtd<br>neiknvwhtnlkkrllksdqsnpnskratkprikrsdsnssiitqsepanlnfremdttsactsssdfssvtvgdsknikcediesletmpvide<br>sfwseaaidetptmssqsltisnemplqypfanyeesfqqshaydsnfddgmdfwtrlgygldtaqdswdtagtqlkmagygsdta

PlMYB122	mvsvnpnpaqgfyffdpsnmalpgvnnlppptptpaapaaapavvedpskkirkpytitksreswteqehdkflealqlfdrdwkkieaf vgsktviqirshaqkyflkvqkngtsehvppprpkrkasrpypqkapktptvsqvmgplqsssafiepayiyspdsssvlgtpvtnmplss wnynttpqpvnvpqvtrddmgltgagqavpnccysssnestpptcpsskrinqgdqgkpikvmpdfaqvysfigsvfdpnstnhlqklr qmdpinvetvlllmrnlsinlmspefedhkrllssydtdsdkskfvnicsksltnkaesavlsa
